# Comparison of growth factor signalling pathway utilisation in cultured normal melanocytes and melanoma cell lines

**DOI:** 10.1186/1471-2407-12-141

**Published:** 2012-04-04

**Authors:** Ji Eun Kim, Clare Stones, Wayne R Joseph, Euphemia Leung, Graeme J Finlay, Andrew N Shelling, Wayne A Phillips, Peter R Shepherd, Bruce C Baguley

**Affiliations:** 1 Auckland Cancer Society Research Centre, The University of Auckland, Auckland, New Zealand; 2 Department of Molecular Medicine and Pathology, The University of Auckland, Auckland, New Zealand; 3 Department of Obstetrics and Gynaecology, The University of Auckland, Auckland, New Zealand; 4 Maurice Wilkins Centre for Molecular Biodiscovery, The University of Auckland, Auckland, New Zealand; 5 Department of Surgery, Surgical Oncology Research Laboratory, Peter MacCallum Cancer Centre and University of Melbourne, St. Vincent's Hospital, Melbourne, VIC, Australia; 6 Auckland Cancer Society Research Centre, Private Bag 92019, The University of Auckland, Auckland, New Zealand

**Keywords:** Phosphatidylinositol-3-kinase, ERK, mTOR, Phosphorylation, Melanoma and Melanocyte

## Abstract

**Background:**

The phosphatidylinositol-3-kinase (PI3K-PKB), mitogen activated protein kinase (MEK-ERK) and the mammalian target of rapamycin (mTOR- p70S6K), are thought to regulate many aspects of tumour cell proliferation and survival. We have examined the utilisation of these three signalling pathways in a number of cell lines derived from patients with metastatic malignant melanoma of known *PIK3CA, PTEN, NRAS *and *BRAF *mutational status.

**Methods:**

Western blotting was used to compare the phosphorylation status of components of the PI3K-PKB, MEK-ERK and mTOR-p70S6K signalling pathways, as indices of pathway utilisation.

**Results:**

Normal melanocytes could not be distinguished from melanoma cells on the basis of pathway utilisation when grown in the presence of serum, but could be distinguished upon serum starvation, where signalling protein phosphorylation was generally abrogated. Surprisingly, the differential utilisation of individual pathways was not consistently associated with the presence of an oncogenic or tumour suppressor mutation of genes in these pathways.

**Conclusion:**

Utilisation of the PI3K-PKB, MEK-ERK and mTOR-p70S6K signalling pathways in melanoma, as determined by phosphorylation of signalling components, varies widely across a series of cell lines, and does not directly reflect mutation of genes coding these components. The main difference between cultured normal melanocytes and melanoma cells is not the pathway utilisation itself, but rather in the serum dependence of pathway utilisation.

## Background

Melanocytes are specialised cells found predominantly in the dermis, hair follicles and eyes, where they have a number of functions including the production of melanin [1] and of other factors including cytokines that act on peripheral cells [2]. Melanomas are thought to arise from excessive proliferation of melanocyte precursors. Melanoma is the most aggressive form of skin cancer that is largely refractory to radiotherapy and cytotoxic drugs and the rapidity of appearance of metastatic lesions also compromises the efficacy of surgery [3].

Growth factor signalling pathways play a key role in relaying extracellular signals from growth factor binding to receptor tyrosine kinases on the plasma membrane to the nucleus via a cascade of phosphorylation events to regulate diverse processes such as proliferation, differentiation, survival and migration in normal melanocytes [4]. The mitogen activated protein kinase (MAPK) signalling cascade is comprised of three-tier kinases that are activated when phosphorylated. The extracellular signal regulated kinase (ERK) pathway is the most studied of the mammalian MAPK pathways and is frequently deregulated in many cancers. ERK1 and ERK2 are activated upon phosphorylation by MEK, which is itself activated when phosphorylated by Raf [5]. The phosphatidylinositol 3-kinase (PI3K) pathway is a second important intracellular signalling pathway and generates phosphatidylinositol-3,4,5-triphosphate (PIP_3_), a second messenger which induces downstream phosphorylation and activation of protein kinase B (PKB also known as Akt). The generation of the second messenger PIP_3 _is antagonised by the tumour suppressor phosphatase and tensin homologue (PTEN) [6]. The mammalian target of rapamycin (mTOR) is a multidomain protein that is related to the PI3K enzymes and mediates signalling to regulate cellular growth and size [7]. Both PI3K and MAPK pathways crosstalk extensively with the mTOR pathway to mediate different cellular functions through two different proteins, ribosomal protein S6 kinase (S6K) and 4E-binding protein (4EBP) [8].

A large fraction of melanomas harbour activating oncogenic or inactivating tumour suppressor gene mutations in the growth factor signalling pathways. Mutations in *BRAF *occur in 40%-60% of melanomas [9,10] and 15%-30% of melanomas harbour activating *NRAS *mutations [10,11]. It is notable that a large percentage of *BRAF *mutant melanomas also contain deletions or mutations in the *PTEN *gene [11]. Although activating mutations of the p110 alpha isoform of PI3K (*PIK3CA*) also contribute to tumourigenesis in many types of cancer [12], they are found only at a low frequency in melanoma [13,14]. However, the activation of the PI3K pathway is more commonly associated with melanoma. In *BRAF *mutant cells, loss of PTEN function plays an important role in the development of melanoma in mouse models, as *BRAF *mutations alone do not induce melanoma but melanoma develops when *PTEN *is deleted in melanocytes which harbour the *BRAF *mutation [15-17]. Current evidence indicates that the PI3K pathway play an important role in melanomas as inhibitors of the PI3K pathway synergise with inhibitors of the MAPK pathway in inhibiting the proliferation of many melanomas [18-20].

The discovery that most human melanomas harbour mutations in either *BRAF *or *NRAS *has led to the development of targeted therapies, such as inhibitors of MEK or BRAF [21]. BRAF inhibitors have been developed that have quite dramatic effects on patients with mutant *BRAF *tumours [22,23]. However responses are followed by the development of resistance [23,24]. Recent studies have outlined the mechanisms whereby melanoma cells acquire resistance by bypassing the signalling pathway that is targeted by the drug. Thus there is a need to understand which signalling pathways are activated in melanoma and how these differ from those used by normal, benign melanocytes.

In an effort to provide a better understanding of the signalling pathways of normal and malignant melanocytes cells, we have cultured samples of surgically resected metastatic melanomas [25] and established over one hundred early passage melanoma cell lines [26-28]. We have analysed these cell lines at early passage for loss of *PTEN *and for mutations in *BRAF, NRAS *and *PIK3CA *and have chosen a subset that is representative of the main patterns of mutation. We have analysed the main signalling pathways of these cell lines and compared them to those of a cell line derived from normal melanocytes. We have characterised the expression and phosphorylation status of the main components of the PI3K and MAPK pathways by western blotting and compared this to gene mutation data. Surprisingly we have found that the pattern of pathway utilisation in normal melanocytes was not distinct from those exhibited by the melanoma lines in the presence of serum. However differences become evident in the absence of serum. Thus, we show that early passage metastatic melanoma cell lines have deregulated growth factor signalling pathways in comparison to primary melanocytes, but that this phenomenon is most clearly manifested upon serum withdrawal.

## Methods

### Culture of melanoma cells and melanocytes

The 12 New Zealand melanoma (NZM) cell lines used for this study were generated from metastatic melanoma after written consent was obtained from all patients under Auckland Area Health Board Ethics Committee guidelines as previously described [27]. NZM cell lines were grown under low oxygen conditions (5% O_2_) in order to mimic physiologically low oxygen levels in tumours. NZM lines were grown in α minimal essential medium (αMEM) (Invitrogen, USA) supplemented with insulin (5 μg/mL), transferrin (5 μg/mL) and sodium selenite (5 ng/mL) (Roche Applied Sciences, Germany), 100 units/mL of penicillin, 100 μg/mL of streptomycin (PS) and 5% fetal bovine serum (FBS). In order to starve cells of serum, culture plates were washed with PBS and incubated with serum free medium (αMEM without FBS and ITS supplement) for 16 hours. Human melanocytes were purchased from Invitrogen and grown in light sensitive Medium 254 supplemented with human melanocyte growth supplement (HMGS-2) (Invitrogen) and PS. Human melanocytes were cultured in an atmosphere of 5% CO_2 _in air at 37°C.

### Genetic analyses of PIK3CA, PTEN, NRAS and BRAF in NZM cell lines

Melanoma cell lines were sequenced for hotspot mutations in *BRAF *exons 11 and 15 and *NRAS *exons 1 and 2. The entire coding region of *PTEN *was also sequenced. The PCR primers for *BRAF *exon 11 were from a published source [9] and the full list of PCR primer sequences are shown in Additional file 1. The PCR reactions were conducted using Taq polymerase, supplemented with BSA to prevent melanin poisoning of Taq polymerase [29].

*BRAF, NRAS *and *PTEN s*equencing reactions were conducted using the PCR primers and sequencing primers that were designed to bind to the PCR product, and run using thermal cycle sequencing with Big Dye Terminator 3.1 chemistry (Applied Biosystems, USA). The reactions were run on a 3130XL Applied Biosystems capillary sequencer (DNA Sequencing Facility, University of Auckland). Mutations were detected manually, using the Codon Code aligner 2.0 programme (CodonCode Corporation), and confirmed by repetition of sequencing from separately amplified material.

Screening for mutations was done in all exons of the *PIK3CA *gene by PCR-single-strand conformational polymorphism (SSCP) as outlined in Campbell *et al*. [30] at the Peter MacCallum Cancer Institute in Melbourne, Australia. Mutations were confirmed by sequencing in both directions.

### Western blotting

After NZM cells were grown to about 80% confluence in the presence of serum or serum starved for 16 hours, they were washed in ice-cold PBS, lysed in radioimmunoprecipitation assay (RIPA) buffer and prepared for western blotting as previously described [31]. Antibodies used were specific for the following epitopes: phosphorylated PKB at Ser473 and Thr308, phosphorylated p70S6K at Thr389, phosphorylated ribosomal protein S6 at Ser240/244 and 235/236, phosphorylated MEK1/2 at Ser217/221 and phosphorylated ERK1/2 at Thr202/Tyr204. Antibodies recognising total PTEN, PKB, p70S6K, rpS6, MEK1/2 and ERK1/2 were also used. All of the above antibodies were from Cell Signaling Technology (USA). β-actin antibody was from Sigma.

## Results

### NZM cell line mutations in the PI3K and MAPK pathways

**Figure 1:**
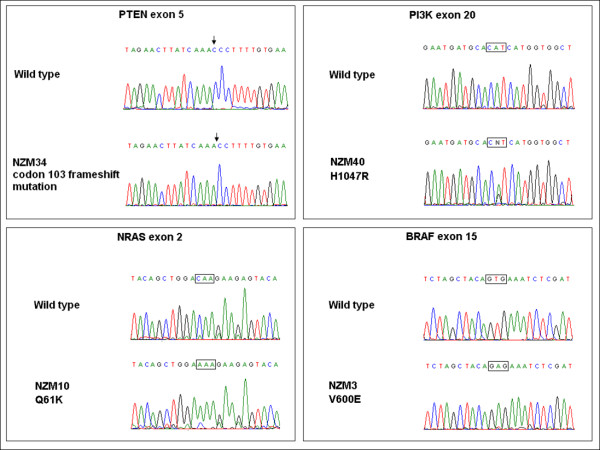
**Examples of sequence electropherograms for wild type (WT) and mutant *PTEN, PIK3CA, NRAS *and *BRAF *genes in selected NZM cell lines**. Black arrows on *PTEN *electropherogram represent the nucleotide deletion resulting in frameshift of the *PTEN *transcript. Nucleotide substitutions in *PIK3CA, NRAS *and *BRAF *are shown in brackets.

**Figure 2:**
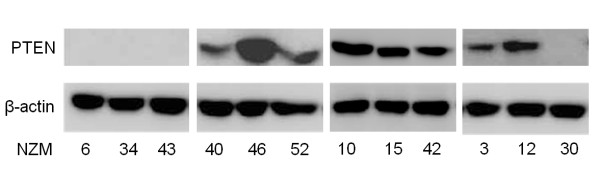
**PTEN protein expression in NZM cell lines**. Equal amounts of protein in whole cell lysates from NZM cell lines were immunoblotted for total PTEN protein expression. β-actin is shown as loading control.

In order to determine whether the presence of activating mutations in the PI3K and MAPK signalling pathways correlated with increased utilisation of downstream signalling pathways, we first determined the mutational status of *PIK3CA, PTEN, NRAS *and *BRAF *genes in the NZM cell line collection. Representative DNA sequences for *PTEN, PIK3CA, BRAF *and *NRAS *are provided in Figure 1. As shown in Table 1, we selected cell lines that were characterised by a number of genetic mutations. All of the selected cell lines harboured either oncogenic V600E or V600K *BRAF *or Q61H *NRAS *mutations. Since the tumour suppressor gene *PTEN *can be functionally lost during melanoma development through both mutation and epigenetic mechanisms [32], we measured PTEN protein expression in the NZM cell lines (Figure 2). Mutation of the *PTEN *gene led to loss of functional PTEN protein expression, as seen in Figure 2. The cell lines NZM40, NZM46 and NZM52, which all harbour the oncogenic H1047R *PIK3CA *mutation, had concurrent *BRAF *or *NRAS *mutations (Table 1). Of particular interest was the high degree of expression of PTEN protein in the NZM46 cell line, compared to other cell lines harbouring the *PIK3CA *oncogenic mutation. Since the presence of an oncogenic mutation or a loss of tumour suppressor function does not dictate whether the cell uses all of the downstream signalling molecules for pathway activation [33,34], we determined the phosphorylation status of the immediate downstream substrates of the PI3K, mTOR and MAPK pathways. Western blots for phosphorylated molecules were used as surrogate markers for pathway activation.

**Table 1:** Mutational status of *PIK3CA, PTEN, NRAS *and *BRAF *genes in New Zealand Melanoma (NZM) cell lines used for the study

Cell lines	*PTEN *status	*PIK3CA *status	*NRAS *status	*BRAF *status
NZM6	Exon3 deletion			V600E

NZM34	Exon 5 frameshift mutation			V600E

NZM43	Exon 1 frameshift mutation			V600K

NZM30	No identified mutation in exon 2-9*			V600E

NZM40		H1047R	Q61H	

NZM46		H1047R	Q61H	

NZM52		H1047R		V600E

NZM10			Q61H	

NZM15			Q61H	

NZM42			Q61H	

NZM3				V600E

NZM12				V600E

*In NZM30, *PTEN *exon 1 sequencing failed after multiple attempts. The absence of PTEN protein in NZM30 (Figure 2) could be due to a large deletion in exon 1 or epigenetic changes

### Phosphorylation of PKB in melanoma and melanocytes

**Figure 3:**
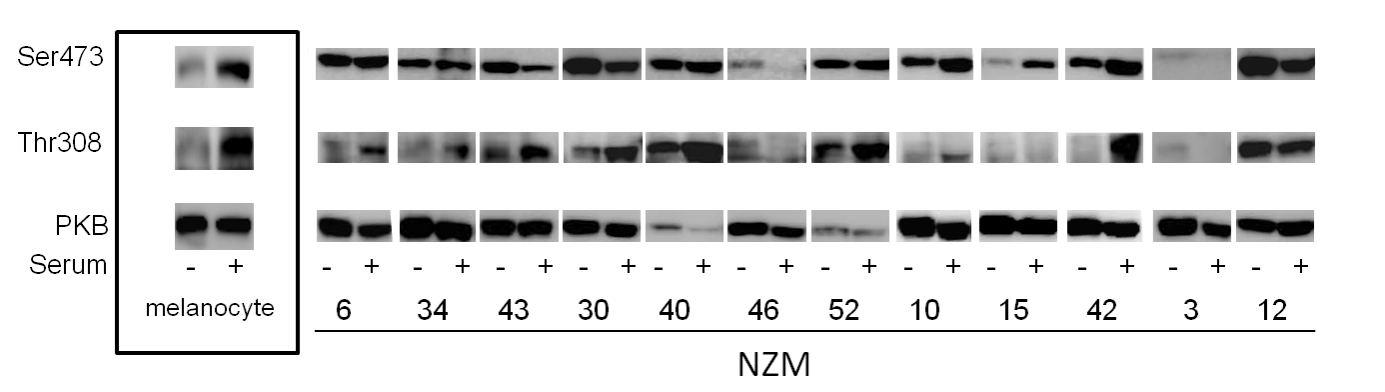
**Phosphorylation of PKB on Ser473 and Thr308 in normal melanocytes and NZM cell lines**. Western blots representative of three independent experiments. Western blots are separated into boxes representing each NZM cell line for clarity. Cells were grown in the absence of FBS for 16 hours (SS) or in the presence of FBS (Ser).

In order to establish whether *PIK3CA, PTEN, NRAS *and *BRAF *mutations resulted in constitutive activation of the downstream signalling pathways, we measured PKB activation by western blotting for phosphorylation at two sites, Ser473 and Thr308. Equal amounts of protein from NZM cell lines were loaded onto the same gel, but for clarity, western blots were segmented to show results for individual NZM cell lines. In melanocytes, phosphorylation of PKB on both Ser473 and Thr308 was strongly serum dependent while most of the NZM cell lines in this study showed serum independent phosphorylation. PKB was phosphorylated independently of serum at the mTORC2 dependent Ser473 site in most of the cell lines, although NZM46 and NZM3 surprisingly had very low levels of phosphorylation even in the presence of serum (Figure 3). In contrast, phosphorylation at the PIP_3_-PDK1 dependent Thr308 site tended to be low in the serum starved state in most cell lines and increased with serum (Figure 3). The notable exceptions were cell lines NZM12, NZM40 and NZM52 which have comparatively high Thr308 phosphorylation in serum starved cells. Phosphorylation of Thr308 in the NZM40 and NZM52 cell lines may be explained by the activating *PIK3CA *mutation in these cells. These two cell lines also have a very low level of total PKB suggesting some feedback regulation of PKB gene expression in these cells. In support of this, NZM46, which also has a *PIK3CA *mutation (Figure 3), also has very high PTEN levels (Figure 2) which could explain the low Thr308 phosphorylation in these cells and the higher levels of total PKB compared to NZM40 and NZM52, as PIP_3 _levels would be predicted to be low despite the *PIK3CA *mutation. NZM46 shows suppression of phosphorylation by serum in the Thr308 site (as with the Ser473 site).

### Phosphorylation of components of the mTOR pathway in melanoma cells and melanocytes

**Figure 4:**
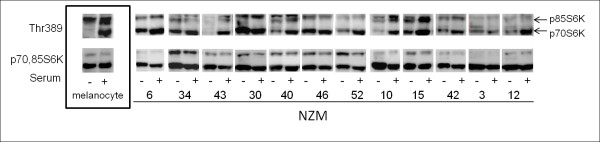
**Phosphorylation of p70S6K and p85S6K on Thr389 in normal melanocytes and NZM cell lines**. The higher molecular weight p85S6K and lower molecular weight p70S6K is indicated by arrows. Western blots representative of three independent experiments. Western blots are separated into boxes representing each NZM cell line for clarity. Cells were grown in the absence of FBS for 16 hours (SS) or in the presence of FBS (Ser).

**Figure 5:**
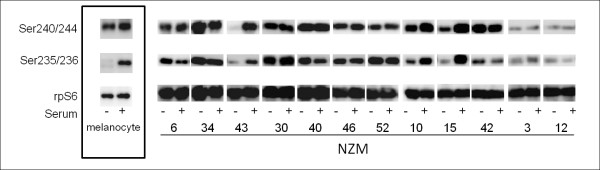
**Phosphorylation of ribosomal protein S6 (rpS6) on Ser240/244 and Ser235/236 in normal melanocytes and NZM cell lines**. Western blots representative of three independent experiments. Western blots are separated into boxes representing each NZM cell line for clarity. Cells were grown in the absence of FBS for 16 hours (SS) or in the presence of FBS (Ser).

Activation of components of the protein translation machinery has been observed in a large percentage of melanomas and is predictive of a poor prognosis [35]. The PI3K signalling pathway can regulate protein translation machinery through mTORC1 and subsequent activation of p70S6K and phosphorylation of ribosomal protein S6 (rpS6). Therefore we next determined the phosphorylation status of p70S6K (Figure 4). The p70S6K was strongly expressed in all cell lines as well as in normal melanocytes but the pattern of phosphorylation of p70S6K and p85S6K at Thr389 did not correlate with the phosphorylation status of PKB nor did it correlate with genotypes (Figure 4). In melanocytes, the observed phosphorylation of Ser235/236 was serum dependent while Ser240/244 site, which is phosphorylated by p70S6K, was phosphorylated even in the absence of serum. In most of the cell lines, we observed serum independent phosphorylation of rpS6 while in NZM43 and to some degree, NZM10 and NZM15 showed serum dependent phosphorylation. Interestingly, we observed little phosphorylation of rpS6 at both sites in *BRAF *mutant cell lines, NZM3 and NZM12 (Figure 5). Thus, phosphorylation of rpS6 is independent of PI3K pathway activation in these melanoma cell lines. In these cells the phosphorylation of rpS6 is likely due to input from the ERK signalling cascade as can be seen in other cell types [36].

### Phosphorylation of components of the ERK pathway in melanoma cells and melanocytes

**Figure 6:**
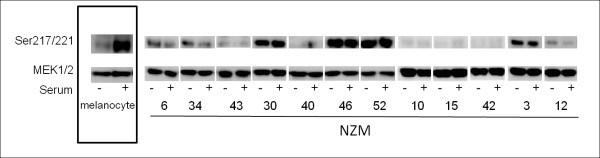
**Phosphorylation of MEK1/2 on Ser217/211 in normal melanocytes and NZM cell lines**. Western blots representative of three independent experiments. Western blots are separated into boxes representing each NZM cell line for clarity. Cells were grown in the absence of FBS for 16 hours (SS) or in the presence of FBS (Ser).

**Figure 7:**
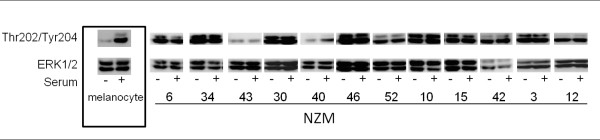
**Phosphorylation of ERK1/2 on Thr202/Tyr204 in normal melanocytes and NZM cell lines**. Western blots representative of three independent experiments. Western blots are separated into boxes representing each NZM cell line for clarity. Cells were grown in the absence of FBS for 16 hours (SS) or in the presence of FBS (Ser).

We also analysed the activation status of the MAPK pathway in NZM cell lines with *NRAS *or *BRAF *mutations and cell lines which additionally harbour *PTEN *or *PIK3CA *mutations. The activation of MEK and then ERK in response to oncogenic *NRAS *and *BRAF *mutations is proposed to be the basis of a MAPK pathway addiction by these cells [37]. Total MEK protein was abundantly expressed in all NZM cell lines as well as melanocytes (Figure 6). However levels of MEK phosphorylation varied considerably and were not directly related to genotype (Figure 6). Furthermore, *NRAS-*only mutant NZM cell lines, NZM10, NZM15 and NZM42 showed very low levels of MEK phosphorylation (Figure 6). ERK was constitutively phosphorylated in almost all cell lines, and unlike melanocytes, NZM cell lines showed serum independent MEK and ERK phosphorylation patterns (Figures 6 and 7). Furthermore, MEK phosphorylation status did not correlate with ERK phosphorylation patterns.

## Discussion

**Figure 8:**
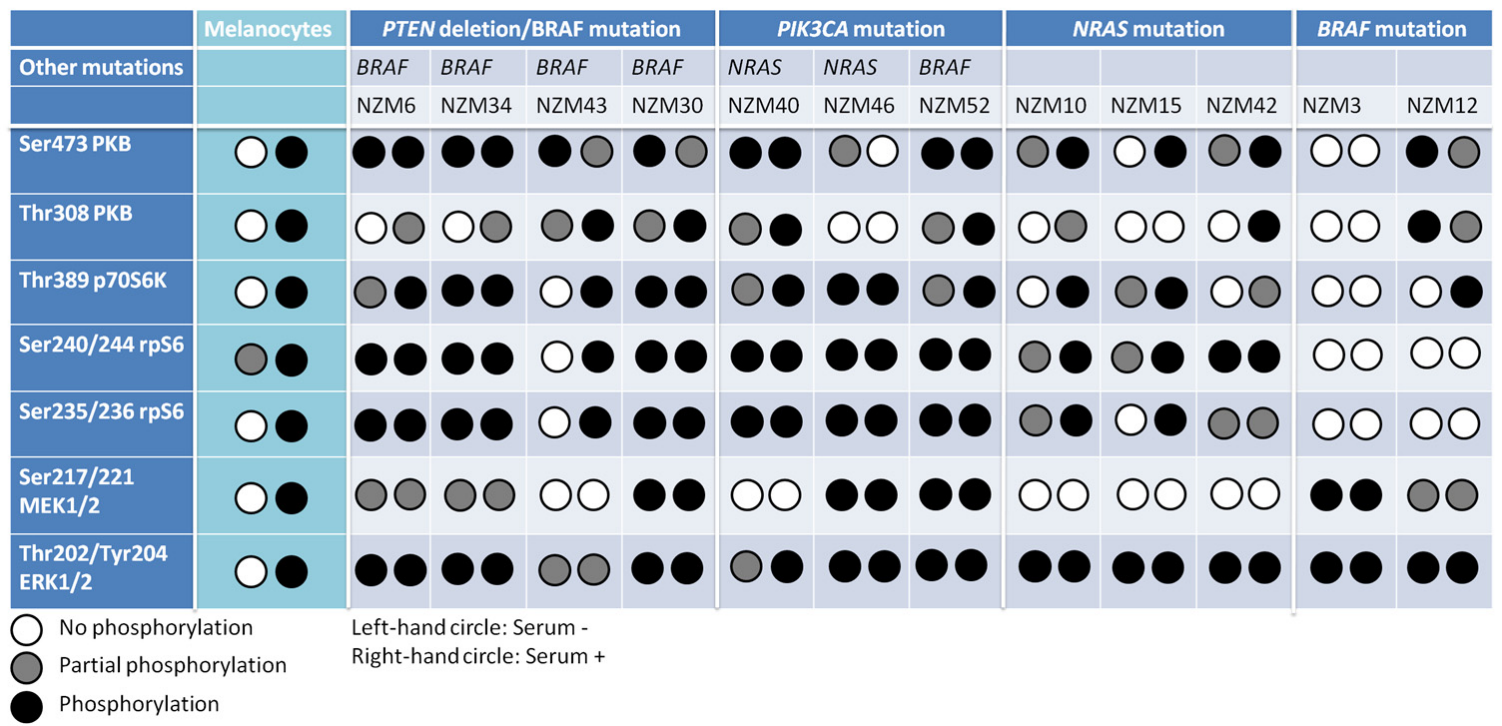
**Summary of Western blotting results in Figures 3, 4, 5, 6 and 7**. Left- and right-hand circles in each cell of the table represent results for cells grown in the absence (SS) and presence (Ser) of FBS.

Traditionally, signal transduction has been described in terms of schematic linear pathways in which stimuli activate or inhibit a series of molecular events that leads to a predictable series of responses. However, recent findings have suggested that signalling occurs in a complex network with extensive cross-talk and context-dependent variations. Signalling pathways also change in response to abnormal proteins arising from mutations and from loss of proteins as a result of epigenetic silencing. Cancer cells are thought to have multiple genetic and epigenetic aberrations, which have complex effects on the circuitry of these signalling networks. Here, in melanocytes and in melanoma cell lines, we have studied the phosphorylation status of key PKB, mTOR and MAPK pathway components downstream of *PTEN, PIK3CA, NRAS *and *BRAF *mutations to determine whether the activity of the signalling pathways correlates with the upstream mutation. In melanocytes, phosphorylation patterns conformed to those expected of the canonical kinase-substrate relationships. Notably, melanocytes showed a consistent serum-dependent phosphorylation status of growth factor signalling pathway proteins. However consistent pattern of phosphorylation was not seen in melanoma cell lines (Figure 8). Our studies are in line with recent findings which indicate that in neoplastic cells, the activity of signalling pathways does not always correlate with the mutational status of upstream proteins especially in the MAPK pathway [34]. This heterogeneity in signalling phenotype is consistent with the high degree of variability in the patterns of gene expression observed in these melanoma cell lines [38,39].

Previous studies have shown that *PIK3CA *mutations can lead to hyperactivated PI3K signalling pathways [40]. However, this phenomenon was not consistently observed in all NZM cell lines studied (Figure 3). Our results are similar to that of Morrows *et al*., [41] who observed different patterns of signalling in colon tumour cell lines harbouring the same mutation. They are also consistent with studies by other groups in a range of non-melanoma cell lines [33,42,43]. A degree of complexity is provided by the results of a recent study of MCF-7 cells [43], in which all of the sublines developed from the parental MCF-7 cell line were all expected to have the same *PIK3CA *mutation, but not all of the sublines showed strong PKB phosphorylation. The results suggest that to some extent the signalling phenotype can be independent of genotype.

All *NRAS*-only mutant cell lines showed serum-independent phosphorylation of ERK1/2 despite no observable phosphorylation of MEK1/2 (Figure 7). The results are surprising but are consistent with the observation of Pratilas *et al*. [44], who found that ERK phosphorylation was not indicative of signalling through the MEK pathway, as ERK phosphorylation is also regulated by negative feedback loops. Furthermore, ERK1/2 is phosphorylated despite little MEK1/2 phosphorylation in some NZM cell lines, suggesting MEK independent regulation of ERK. It has been suggested that PI3K and classical protein kinase C (cPKC) play a major role in the MEK-independent prolonged activation of ERK in some cell types [45,46]. As all the NZM cell lines used in this study are mutant for either *BRAF *or *NRAS*, this suggests that these oncogenic mutations confer activation of the MAPK pathway. However, the dominant signalling pattern observed in all of the NZM cell lines is serum independent phosphorylation of ERK1/2 compared to melanocytes. We also did not observe NZM cell lines lacking *PTEN *function to be strongly associated with inactivation of MEK1/2 and ERK1/2 in the MAPK pathway as noted by Dan *et al*. (2010) [47]. A possible explanation for this is that all of the NZM cell lines studied for functional *PTEN *loss also have *BRAF *mutations. Although Dan *et al*. [47] suggests that mutations in either *NRAS *or *BRAF *are strongly correlated with PI3K-PKB pathway inactivation, we did not observe this in the panel of NZM cell lines.

A further result of this study is that, in the presence of serum, the phosphorylation pattern of normal melanocytes is generally similar to that of melanoma cells; differences are more clearly seen when the cell lines are grown in the absence of serum. Unlike melanocytes, melanoma cells are frequently serum independent, may show low phosphorylation in the presence of serum and may show suppression of phosphorylation by the addition of serum. It might be argued that the addition of serum, by stimulating multiple signalling pathways linked to growth factor receptors on the plasma membrane, obscures the signalling pattern derived from an activated component such as PI3K or BRAF, but the data from serum-starved cultures did not provide any clear relationship between mutational status and pathway utilisation. Further experiments with specific inhibitors of these pathways, such as PI3K, PKB, MEK and mTOR prevented phosphorylation of the corresponding downstream target (data not shown), indicating that actions of inhibitors does not depend on activation of upstream signalling molecules. The difference in the dependence of melanocytes and melanoma cells on serum growth factors for phosphorylation of downstream signalling molecules could be due to autocrine growth factors produced in melanomas. It has been noted that melanomas produce vascular endothelial growth factor (VEGF) [48,49] and fibroblast growth factor (FGF) [50], which could explain this loss of serum dependence. Melanomas may also over-express growth factor receptors such as insulin-like growth factor 1 receptor (IGF1-R) [51] and Axl [52] which can support constitutive activation of some components of the growth factor pathway.

## Conclusion

In conclusion, we found that activation of the growth factor signalling pathways varied considerably among a series of NZM cell lines, and that no consistent relationship was observed between pathway activation, as measured by protein phosphorylation. However despite this heterogeneity, there was clearly an observable difference between melanoma cells and normal melanocytes upon serum starvation in growth factor signalling pathways amongst the NZM cell lines. Therefore, the main difference found between normal melanocytes and melanoma cells in culture was the serum dependence of pathway utilisation. Although the sensitivity of the cells harbouring different mutations to inhibitors of the PI3K and MAPK pathways is currently being investigated, unpredictable signalling activation patterns observed in response to mutations suggest that sensitivity to inhibitors between cell lines harbouring the same mutation may be highly variable. Our findings in cultured melanoma cells suggest that the presence of activated PI3K or BRAF does induce consistent, albeit unexpected changes in global cellular signalling. Also, it is possible that different signals arising from mutations in other pathways can crosstalk with the studied pathways to produce unpredictable responses as we have observed. Microenvironmental influences (such as paracrine signalling) may alter the utilisation of a certain signalling pathway over another. Although we measured phosphorylation status as readout for signalling pathway activation, a more comprehensive analysis of downstream signalling pathways such as transcriptional readout [44] and analysis of the proliferation of cell lines in response to various inhibitors [20] is expected to give a better understanding of growth factor signalling pathways in melanoma. Moreover, epigenetic regulation may play a greater part in dictating pathway activation independent of activating oncogenes or loss of tumour suppressor mutations, which will produce heterogeneity.

## Competing interests

The authors declare that they have no competing interests.

## Authors' contributions

JK performed all the western blot experiments and CS performed the sequencing analysis. WRJ, EL, GJF, ANS, WAP, PRS contributed reagents. JK and BCB wrote the manuscript with revision from GJF, ANS, WAP and PRS. All authors read and approved the final manuscript.

## Pre-publication history

The pre-publication history for this paper can be accessed here:

http://www.biomedcentral.com/1471-2407/12/141/prepub

## Supplementary Material

Additional file 1 **Table S1 PCR primer and sequencing primer sequences used for the study**. The Reference sequences (NCBI) for *PTEN*-NT_030059.12, *PIK3CA*-NT_000003.11, *NRAS*-NC_000001.10 and *BRAF*-NC_000007.13. ^# ^The *BRAF *exon 11 PCR primers were taken from Davies et al. [9]. *The primers for exon 9-13 of *PIK3CA *were designed to not match a pseudogene on chromosome 22 [53]. Click here for file

## References

[B1] Gray-Schopfer V Wellbrock C Marais R Melanoma biology and new targeted therapy Nature 2007 445 7130 851 857 10.1038/nature05661 17314971

[B2] Tsatmali M Ancans J Thody AJ Melanocyte function and its control by melanocortin peptides J Histochem Cytochem 2002 50 2 125 133 10.1177/002215540205000201 11799132

[B3] Russo AE Torrisi E Bevelacqua Y Perrotta R Libra M McCubrey JA Spandidos DA Stivala F Malaponte G Melanoma: molecular pathogenesis and emerging target therapies (Review) Int J Oncol 2009 34 6 1481 1489 19424565 10.3892/ijo_00000277

[B4] Inamdar GS Madhunapantula SV Robertson GP Targeting the MAPK pathway in melanoma: why some approaches succeed and other fail Biochem Pharmacol 2010 80 5 624 637 10.1016/j.bcp.2010.04.029 20450891 PMC2897908

[B5] Dhillon AS Hagan S Rath O Kolch W MAP kinase signalling pathways in cancer Oncogene 2007 26 22 3279 3290 10.1038/sj.onc.1210421 17496922

[B6] Leslie NR Downes CP PTEN function: how normal cells control it and tumour cells lose it Biochem J 2004 382 Pt 1 1 11 15193142 10.1042/BJ20040825 PMC1133909

[B7] Wang X Proud CG The mTOR pathway in the control of protein synthesis Physiology (Bethesda) 2006 21 362 369 10.1152/physiol.00024.2006 16990457

[B8] Choo AY Yoon SO Kim SG Roux PP Blenis J Rapamycin differentially inhibits S6Ks and 4E-BP1 to mediate cell-type-specific repression of mRNA translation Proc Natl Acad Sci USA 2008 105 45 17414 17419 10.1073/pnas.0809136105 18955708 PMC2582304

[B9] Davies H Bignell GR Cox C Stephens P Edkins S Clegg S Teague J Woffendin H Garnett MJ Bottomley W Mutations of the BRAF gene in human cancer Nature 2002 417 6892 949 954 10.1038/nature00766 12068308

[B10] Omholt K Platz A Kanter L Ringborg U Hansson J NRAS and BRAF Mutations Arise Early during Melanoma Pathogenesis and Are Preserved throughout Tumor Progression Clin Cancer Res 2003 9 17 6483 6488 14695152

[B11] Tsao H Goel V Wu H Yang G Haluska FG Genetic interaction between NRAS and BRAF mutations and PTEN/MMAC1 inactivation in melanoma J Invest Dermatol 2004 122 2 337 341 10.1046/j.0022-202X.2004.22243.x 15009714 PMC2586668

[B12] Wong K-K Engelman JA Cantley LC Targeting the PI3K signaling pathway in cancer Curr Opin Genet Dev 2010 20 1 87 90 10.1016/j.gde.2009.11.002 20006486 PMC2822054

[B13] Board RE Thelwell NJ Ravetto PF Little S Ranson M Dive C Hughes A Whitcombe D Multiplexed assays for detection of mutations in PIK3CA Clin Chem 2008 54 4 757 760 10.1373/clinchem.2007.098376 18375489

[B14] Hafner C Landthaler M Vogt T Activation of the PI3K/AKT signalling pathway in non-melanoma skin cancer is not mediated by oncogenic PIK3CA and AKT1 hotspot mutations Exp Dermatol 2010 19 8 e222 e227 10.1111/j.1600-0625.2009.01056.x 20557351

[B15] Babchia N Calipel A Mouriaux F Faussat A-M Mascarelli F The PI3K/Akt and mTOR/P70S6K signaling pathways in human uveal melanoma cells: interaction with B-Raf/ERK Investig Ophthalmol Vis Sci 2010 51 1 421 429 10.1167/iovs.09-3974 19661225

[B16] Dankort D Curley DP Cartlidge RA Nelson B Karnezis AN Damsky WE Jr You MJ DePinho RA McMahon M Bosenberg M Braf(V600E) cooperates with Pten loss to induce metastatic melanoma Nat Genet 2009 41 5 544 552 10.1038/ng.356 19282848 PMC2705918

[B17] Stahl JM Cheung M Sharma A Trivedi NR Shanmugam S Robertson GP Loss of PTEN promotes tumor development in malignant melanoma Cancer Res 2003 63 11 2881 2890 12782594

[B18] Gopal YN Deng W Woodman SE Komurov K Ram P Smith PD Davies MA Basal and treatment-induced activation of AKT mediates resistance to cell death by AZD6244 (ARRY-142886) in Braf-mutant human cutaneous melanoma cells Cancer Res 2010 70 21 8736 8747 10.1158/0008-5472.CAN-10-0902 20959481 PMC4286702

[B19] Madhunapantula SV Robertson GP The PTEN-AKT3 signaling cascade as a therapeutic target in melanoma Pigment Cell Melanoma Res 2009 22 4 400 419 10.1111/j.1755-148X.2009.00585.x 19493313 PMC3610526

[B20] Jiang CC Lai F Thorne RF Yang F Liu H Hersey P Zhang XD MEK-Independent Survival of B-RAFV600E Melanoma Cells Selected for Resistance to Apoptosis Induced by the RAF Inhibitor PLX4720 Clin Cancer Res 2011 17 4 721 730 10.1158/1078-0432.CCR-10-2225 21088259

[B21] Flaherty KT McArthur G BRAF, a target in melanoma: implications for solid tumor drug development Cancer 2010 116 21 4902 4913 10.1002/cncr.25261 20629085

[B22] Flaherty KT Puzanov I Kim KB Ribas A McArthur GA Sosman JA O'Dwyer PJ Lee RJ Grippo JF Nolop K Inhibition of mutated, activated BRAF in metastatic melanoma N Engl J Med 2010 363 9 809 819 10.1056/NEJMoa1002011 20818844 PMC3724529

[B23] Nazarian R Shi H Wang Q Kong X Koya RC Lee H Chen Z Lee M-K Attar N Sazegar H Melanomas acquire resistance to B-RAF(V600E) inhibition by RTK or N-RAS upregulation Nature 2010 468 7326 973 977 10.1038/nature09626 21107323 PMC3143360

[B24] Johannessen CM Boehm JS Kim SY Thomas SR Wardwell L Johnson LA Emery CM Stransky N Cogdill AP Barretina J COT drives resistance to RAF inhibition through MAP kinase pathway reactivation Nature 2010 468 7326 968 972 10.1038/nature09627 21107320 PMC3058384

[B25] Marshall ES Finlay GJ Matthews JH Shaw JH Nixon J Baguley BC Microculture-based chemosensitivity testing: a feasibility study comparing freshly explanted human melanoma cells with human melanoma cell lines J Natl Cancer Inst 1992 84 5 340 345 10.1093/jnci/84.5.340 1738186

[B26] Charters GA Stones CJ Shelling AN Baguley BC Finlay GJ Centrosomal dysregulation in human metastatic melanoma cell lines Cancer Genetics 2011 204 9 477 485 10.1016/j.cancergen.2011.07.001 22018269

[B27] Marshall ES Matthews JH Shaw JH Nixon J Tumewu P Finlay GJ Holdaway KM Baguley BC Radiosensitivity of new and established human melanoma cell lines: comparison of [3 H]thymidine incorporation and soft agar clonogenic assays Eur J Cancer 1994 30A 9 1370 1376 7999427 10.1016/0959-8049(94)90188-0

[B28] Parmar J Marshall ES Charters GA Holdaway KM Shelling AN Baguley BC Radiation-induced cell cycle delays and p53 status of early passage melanoma cell lines Oncol Res 2000 12 3 149 155 11216673 10.3727/096504001108747620

[B29] Giambernardi TA Rodeck U Klebe RJ Bovine serum albumin reverses inhibition of RT-PCR by melanin Biotechniques 1998 25 4 564 566 9793633 10.2144/98254bm03

[B30] Campbell IG Russell SE Choong DYH Montgomery KG Ciavarella ML Hooi CSF Cristiano BE Pearson RB Phillips WA Mutation of the PIK3CA gene in ovarian and breast cancer Cancer Res 2004 64 21 7678 7681 10.1158/0008-5472.CAN-04-2933 15520168

[B31] Kim JE Shepherd PR Chaussade C Investigating the role of class-IA PI 3-kinase isoforms in adipocyte differentiation Biochem Biophys Res Commun 2009 379 4 830 834 10.1016/j.bbrc.2008.12.089 19114029

[B32] Mirmohammadsadegh A Marini A Nambiar S Hassan M Tannapfel A Ruzicka T Hengge UR Epigenetic silencing of the PTEN gene in melanoma Cancer Res 2006 66 13 6546 6552 10.1158/0008-5472.CAN-06-0384 16818626

[B33] Vasudevan KM Barbie DA Davies MA Rabinovsky R McNear CJ Kim JJ Hennessy BT Tseng H Pochanard P Kim SY AKT-independent signaling downstream of oncogenic PIK3CA mutations in human cancer Cancer Cell 2009 16 1 21 32 10.1016/j.ccr.2009.04.012 19573809 PMC2752826

[B34] Houben R Vetter-Kauczok CS Ortmann S Rapp UR Broecker EB Becker JC Phospho-ERK Staining Is a Poor Indicator of the Mutational Status of BRAF and NRAS in Human Melanoma J Invest Dermatol 2008 128 8 2003 2012 10.1038/jid.2008.30 18323787

[B35] O'Reilly KE Warycha M Davies MA Rodrik V Zhou XK Yee H Polsky D Pavlick AC Rosen N Bhardwaj N Phosphorylated 4E-BP1 is associated with poor survival in melanoma Clin Cancer Res 2009 15 8 2872 2878 10.1158/1078-0432.CCR-08-2336 19336517 PMC3995540

[B36] Wang L Gout I Proud CG Cross-talk between the ERK and p70 S6 kinase (S6K) signaling pathways. MEK-dependent activation of S6K2 in cardiomyocytes J Biol Chem 2001 276 35 32670 32677 10.1074/jbc.M102776200 11431469

[B37] Weinstein IB Joe A Oncogene addiction Cancer Res 2008 68 9 3077 3080 discussion 3080 10.1158/0008-5472.CAN-07-3293 18451130

[B38] Jeffs AR Glover AC Slobbe LJ Wang L He S Hazlett JA Awasthi A Woolley AG Marshall ES Joseph WR A gene expression signature of invasive potential in metastatic melanoma cells PLoS One 2009 4 12 e8461 10.1371/journal.pone.0008461 20041153 PMC2794539

[B39] Quintana E Shackleton M Foster HR Fullen DR Sabel MS Johnson TM Morrison SJ Phenotypic heterogeneity among tumorigenic melanoma cells from patients that is reversible and not hierarchically organized Cancer Cell 2010 18 5 510 523 10.1016/j.ccr.2010.10.012 21075313 PMC3031091

[B40] Samuels Y Diaz LA Jr Schmidt-Kittler O Cummins JM Delong L Cheong I Rago C Huso DL Lengauer C Kinzler KW Mutant PIK3CA promotes cell growth and invasion of human cancer cells Cancer Cell 2005 7 6 561 573 10.1016/j.ccr.2005.05.014 15950905

[B41] Morrow CJ Gray A Dive C Comparison of phosphatidylinositol-3-kinase signalling within a panel of human colorectal cancer cell lines with mutant or wild-type PIK3CA FEBS Lett 2005 579 23 5123 5128 10.1016/j.febslet.2005.07.096 16150444

[B42] Torbett NE Luna-Moran A Knight ZA Houk A Moasser M Weiss W Shokat KM Stokoe D A chemical screen in diverse breast cancer cell lines reveals genetic enhancers and suppressors of sensitivity to PI3K isoform-selective inhibition Biochem J 2008 415 1 97 110 10.1042/BJ20080639 18498248 PMC3079392

[B43] Leung E Kannan N Krissansen GW Findlay MP Baguley BC MCF-7 breast cancer cells selected for tamoxifen resistance acquire new phenotypes differing in DNA content, phospho-HER2 and PAX2 expression, and rapamycin sensitivity Cancer Biol Ther 2010 9 9 717 724 10.4161/cbt.9.9.11432 20234184

[B44] Pratilas CA Taylor BS Ye Q Viale A Sander C Solit DB Rosen N V600EBRAF is associated with disabled feedback inhibition of RAF-MEK signaling and elevated transcriptional output of the pathway Proc Natl Acad Sci 2009 106 11 4519 4524 10.1073/pnas.0900780106 19251651 PMC2649208

[B45] Aksamitiene E Kholodenko BN Kolch W Hoek JB Kiyatkin A PI3K/Akt-sensitive MEK-independent compensatory circuit of ERK activation in ER-positive PI3K-mutant T47D breast cancer cells Cell Signal 2010 22 9 1369 1378 10.1016/j.cellsig.2010.05.006 20471474 PMC2893265

[B46] Grammer TC Blenis J Evidence for MEK-independent pathways regulating the prolonged activation of the ERK-MAP kinases Oncogene 1997 14 14 1635 1642 10.1038/sj.onc.1201000 9135064

[B47] Dan S Okamura M Seki M Yamazaki K Sugita H Okui M Mukai Y Nishimura H Asaka R Nomura K Correlating phosphatidylinositol 3-kinase inhibitor efficacy with signaling pathway status: in silico and biological evaluations Cancer Res 2010 70 12 4982 4994 10.1158/0008-5472.CAN-09-4172 20530683

[B48] Graells J Vinyals A Figueras A Llorens A Moreno A Marcoval J Gonzalez F Fabra A Overproduction of VEGF165 Concomitantly Expressed with its Receptors Promotes Growth and Survival of Melanoma Cells through MAPK and PI3K Signaling J Investig Dermatol 2004 123 6 1151 1161 10.1111/j.0022-202X.2004.23460.x 15610528

[B49] Lacal PM Ruffini F Pagani E D'Atri S An autocrine loop directed by the vascular endothelial growth factor promotes invasiveness of human melanoma cells Int J Oncol 2005 27 6 1625 1632 16273219

[B50] Lefèvre G Babchia N Calipel A Mouriaux F Faussat A-M Mrzyk S Mascarelli F Activation of the FGF2/FGFR1 autocrine loop for cell proliferation and survival in uveal melanoma cells Investig Ophthalmol Vis Sci 2009 50 3 1047 1057 19029025 10.1167/iovs.08-2378

[B51] Kanter-Lewensohn L Dricu A Girnita L Wejde J Larsson O Expression of insulin-like growth factor-1 receptor (IGF-1R) and p27Kip1 in melanocytic tumors: a potential regulatory role of IGF-1 pathway in distribution of p27Kip1 between different cyclins Growth Factors 2000 17 3 193 202 10.3109/08977190009001068 10705577

[B52] Shearer RL Van Ginkel PR Polans AS Over-expression of the receptor tyrosine kinase axl promotes ocular melanoma cell survival Invest Ophthalmol Vis Sci 2002 43 12 1125 10.1158/0008-5472.can-03-0245 14729616

[B53] Campbell IG Russell SE Phillips WA PIK3CA mutations in ovarian cancer Clin Cancer Res 2005 11 19 7042 7043 10.1158/1078-0432.CCR-05-1024 16203798

