# Supplementary material for: Comparison of growth factor signalling pathway utilisation in cultured normal melanocytes and melanoma cell lines
Source: BMC Cancer. 2012 Apr 4;12:141. doi: 10.1186/1471-2407-12-141 (PMC3352269; doi:10.1186/1471-2407-12-141)
Supplement: Additional file 1 — Table S1 PCR primer and sequencing primer sequences used for the study. The Reference sequences (NCBI) for PTEN-NT_030059.12, PIK3CA-NT_000003.11, NRAS-NC_000001.10 and BRAF-NC_000007.13. # The BRAF exon 11 PCR primers were taken from Davies et al. [9]. *The primers for exon 9-13 of PIK3CA were designed to not match a pseudogene on chromosome 22 [53]. [file 1471-2407-12-141-S1.DOC]

| **Gene and exon** | **Primers** | **Primer sequence** |
| --- | --- | --- |
| *BRAF* exon 11 | Forward^#^ | TCCCTCTCAGGCATAAGGTAA |
|  | Reverse^#^ | CGAACAGTGAATATTTCCTTTGAT |
|  | Sequencing | CGAACAGTGAATATTTCCTTTGATGAT |
| *BRAF* exon 15 | Forward | CACCTCATCCTAACACATTTCAAG |
|  | Reverse | TTTCAACAGGGTACACAGAACAT |
| *NRAS* exon 1 | Forward | ATTAATCCGGTGTTTTTGCGTTCT |
|  | Reverse | CATCTCTGAATCCTTTATCTCCAT |
| *NRAS* exon 2 | Forward | AACAGCACAAATAAAACAGTCCAG |
|  | Reverse | GGTTCCAAGTCATTCCCAGTA |
| *PTEN* exon 1 | Forward | GCCTCCTCTTCGTCTTTTCTAACC |
|  | Reverse | TATATGACCTAGCAACCTGACCA |
|  | Sequencing | CTCTCCCCTTCTACTGCCTCCA |
| *PTEN* exon 2 | Forward | TTAGCCATGAAAAATTAGAAGTTG |
|  | Reverse | CTGTATCCCCCTGAAGTCCA |
| *PTEN* exon 3 | Forward | GGGGTATTTGTTGGATTATTTATT |
|  | Reverse | GCCCTAACAGCTTTTTCAGTCA |
| *PTEN* exon 4 | Forward | GTAAGCATTTGGCCAAGTAGATT |
|  | Reverse | AGTAGTTCTGCTTTCTCCCTGTG |
|  | Sequencing | TTTGAAAGCATGGAAGCACCTG |
| *PTEN* exon 5 | Forward | TTGGAATATGTTTTGTGATGATGA |
|  | Reverse | GGAGGGAGGAACACAAGATGAA |
|  | Sequencing | TTACTTGTCAATTACACCTCAATAAAACTG |
| *PTEN* exon 6 | Forward | GCCTTTGCCTATGGGGTTCA |
|  | Reverse | ATTGGGCTGTATTTGGTGGTTAT |
| *PTEN* exon 7 | Forward | TTTCGTGACTACTCCCTGTTGATA |
|  | Reverse | TTGGTCCCATGCTAATTTCTTCTC |
| *PTEN* exon 8 | Forward | TGAATGAAAATGCAACAGATAACT |
|  | Reverse | AATAAATTGGAAGGCAGACAGGAG |
| *PTEN* exon 9 | Forward | ACCTAGCAAGAAAGAAAATGTTGA |
|  | Reverse | AAAACTGGAATAAAACGGGAAAGT |
| *PIK3CA* exon 1 | Forward | ACAACCATACATCTAATTCCTTAAAGT |
|  | Reverse | GCTTTATGGTTATTTGCATTTTAGA |
| *PIK3CA* exon 2 | Forward | TTCATGCTGTGTATGTAATAGAATGTT |
|  | Reverse | AGACACAGGTAGAAGACTGCACTA |
| *PIK3CA* exon 3 | Forward | TGAAAGAGAGATGGTGATTGC |
|  | Reverse | CAGATACTCATCCTCAATGTGATT |
| *PIK3CA* exon 4 | Forward | TGAAAAACCTTACAGGAAATGG |
|  | Reverse | AGTGCAAGAAAAAGGTTATCTAAAA |
| *PIK3CA* exon 5 | Forward | CGAGTGTGTGCATATGTGTATGTT |
|  | Reverse | GCCCAGGCTGGTCTAAAAA |
| *PIK3CA* exon 6 | Forward | TCTTTGTCTTCGTGATTTGTAGGA |
|  | Reverse | TCAATCAGCGGTATAATCAGGA |
| *PIK3CA* exon 7 | Forward | CCTTTTGGGGAAGAAAAGTG |
|  | Reverse | GAGAGAAGGTTTGACTGCCATAA |
| *PIK3CA* exon 8 | Forward | TTTTTATGGCAGTCAAACCTTC |
|  | Reverse | GAGAAAGTATCTACCTAAATCCACA |
| *PIK3CA* exon 9* | Forward | TGAAAATGTATTTGCTTTTTCTGT |
|  | Reverse | TGTAAATTCTGCTTTATTTATTCC |
| *PIK3CA* exon 10* | Forward | ACCTTTTGAACAGCATGCAA |
|  | Reverse | TTCTGAGAGAAAACAATTTAAGTGA |
| *PIK3CA* exon 11* | Forward | GGCAGTGTTTTAGATGGCTCA |
|  | Reverse | AAAAGGAAGTTGTATGGATCTAG |
| *PIK3CA* exon 12* | Forward | CGGCCATGCAGAAACTGAC |
|  | Reverse | TGTACCTTAAGAATTTAATGGGAAAA |
| *PIK3CA* exon 13* | Forward | ATATATTTTTAATTTTGCACGATTC |
|  | Reverse | GTGAAAAGAGTCTCAAACACAA |
| *PIK3CA* exon 14 | Forward | TCTCATGTGAGAAAGAGATTAGCA |
|  | Reverse | TTCTCAAGATTTTATCCAGAAAAGG |
| *PIK3CA* exon 15 | Forward | CCTAAATAAAAATTGAGGTGAAAGT |
|  | Reverse | TGGCTTTCAGTAGTTTTCATGG |
| *PIK3CA* exon 16 | Forward | AAACCATGTGATGGCGTGAT |
|  | Reverse | CACTTTTTCAGGATAACTTTCAACA |
| *PIK3CA* exon 17 | Forward | GCCTGTTAAAACATTTGCTATTTT |
|  | Reverse | CCGACAGACTCATCTAACAAAAAC |
| *PIK3CA* exon 18 | Forward | TGGAAACTTGCACCCTGTTT |
|  | Reverse | TGCAGATACAAAATGTCTTGAATAA |
| *PIK3CA* exon 19 | Forward | TCTCTGTTTAAAATGTTTTGGTGTT |
|  | Reverse | GGGATTACAGGCATGAACCA |
| *PIK3CA* exon 20 | Forward | CATTTGCTCCAAACTGACCA |
|  | Reverse | GGTCTTTGCCTGCTGAGAGT |
